# The Relationship between Placental Shear Wave Elastography and Fetal Weight—A Prospective Study

**DOI:** 10.3390/jcm13154432

**Published:** 2024-07-29

**Authors:** Erika Cavanagh, Kylie Crawford, Jesrine Gek Shan Hong, Davide Fontanarosa, Christopher Edwards, Marie-Luise Wille, Jennifer Hong, Vicki L. Clifton, Sailesh Kumar

**Affiliations:** 1 Mater Research Institute, University of Queensland, Level 3, Aubigny Place, Raymond Terrace, South Brisbane, QLD 4101, Australia; ej1.robinson@hdr.qut.edu.au (E.C.); kylie.crawford@mater.uq.edu.au (K.C.); jesrine.hong@uq.edu.au (J.G.S.H.); vicki.clifton@mater.uq.edu.au (V.L.C.); 2 School of Clinical Sciences, Faculty of Health, Queensland University of Technology, Brisbane, QLD 4000, Australiac8.edwards@qut.edu.au (C.E.); 3 Centre for Biomedical Technologies (CBT), Queensland University of Technology, Brisbane, QLD 4000, Australia; 4 Mater Centre for Maternal and Fetal Medicine, Mater Mother’s Hospital, South Brisbane, QLD 4101, Australia; jennifer.hong@mater.org.au; 5 Department of Obstetrics and Gynecology, Faculty of Medicine, Universiti Malaya, Kuala Lumpur 50603, Malaysia; 6 School of Mechanical, Medical and Process Engineering and ARC Training Centre for Multiscale 3D Imaging, Modelling and Manufacturing, Queensland University of Technology, Brisbane, QLD 4000, Australia; m.wille@qut.edu.au; 7 Faculty of Medicine, The University of Queensland, Herston, QLD 4006, Australia

**Keywords:** shear wave elastography, shear wave velocity, placental function, placental stiffness, fetal growth, fetal growth restriction, small for gestational age, ultrasound

## Abstract

**Background/Objectives**: The utility of shear wave elastography (SWE) as an adjunct to ultrasound biometry and Doppler velocimetry for the examination of placental dysfunction and suboptimal fetal growth is unclear. To date, limited data exist correlating the mechanical properties of placentae with fetal growth. This study aimed to investigate the relationship between placental shear wave velocity (SWV) and ultrasound estimated fetal weight (EFW), and to ascertain if placental SWV is a suitable proxy measure of placental function in the surveillance of small-for-gestational-age (SGA) pregnancies. **Methods**: This prospective, observational cohort study compared the difference in placental SWV between SGA and appropriate-for-gestational-age (AGA) pregnancies. There were 221 women with singleton pregnancies in the study cohort—136 (61.5%) AGA and 85 (38.5%) SGA. Fetal biometry, Doppler velocimetry, the deepest vertical pocket of amniotic fluid, and mean SWV were measured at 2–4-weekly intervals from recruitment to birth. **Results**: There was no difference in mean placental SWV in SGA pregnancies compared to AGA pregnancies, nor was there any relationship to EFW. **Conclusions**: Although other studies have shown some correlation between increased placental stiffness and SGA pregnancies, our investigation did not support this. The mechanical properties of placental tissue in SGA pregnancies do not result in placental SWVs that are apparently different from those of AGA controls. As this study did not differentiate between constitutionally or pathologically small fetuses, further studies in growth-restricted cohorts would be of benefit.

## 1. Introduction

Suboptimal fetal growth resulting in a small-for-gestational-age (SGA) or low-birth-weight infant is a leading cause of severe neonatal morbidity and mortality globally [1,2,3,4]. These small and vulnerable newborns are also at a disproportionately higher risk of developing chronic non-communicable diseases in childhood, adolescence, and adulthood [5,6]. It is now well established that the placenta is central to the etiology of pre-eclampsia and fetal growth restriction (FGR) [6]. Deficiencies in placentation in early pregnancy (evidenced by imaging, histological, and biomarker data) have been shown to lead to chronic malperfusion, resulting in or contributing to the maternal syndrome and/or suboptimal fetal growth [6,7,8,9]. Histological analysis in particular shows evidence of maternal vascular malperfusion, spiral artery atherosis, and oxidative stress secondary to ischemia–reperfusion of the placental villous tree, resulting in mechanical damage, a reduction in placental volume and surface area, and increased vascular resistance in the maternal–fetal circulation [6,10].

The diagnosis, surveillance, and management of suspected placental dysfunction, particularly in early gestation, remains a major challenge for obstetricians [11]. Although the assessment of fetal wellbeing using fetal biometry and Doppler velocimetry of fetoplacental vessels identifies some infants at risk of death and severe morbidity, these indirect assessments of placental function are not always sufficiently reliable, thus limiting the ability to mitigate adverse perinatal outcomes [6,12,13,14].

Mechanical properties of biological tissues are regulated by their viscoelasticity [15]. Non-invasive assessment techniques can be used to evaluate changes in tissue stiffness in certain pathological conditions [16,17]. One such assessment technique, shear wave elastography (SWE), is a novel ultrasound-derived method for estimating tissue stiffness and can be used in conjunction with conventional ultrasound to objectively quantify inflammatory changes and tissue fibrosis, as well as differentiate between healthy and diseased tissue [15,18,19,20]. This non-invasive and reproducible technique utilizes a modified acoustic wave of higher intensity than conventional diagnostic ultrasound to produce lateral shear waves perpendicular to the plane of the ultrasound beam. These shear waves deform the tissue within the organ of interest, and the velocity of the shear waves propagating away from the pulse can be quantified [20]. The measured velocity of the shear wave is proportionate to the stiffness of the tissue, with a higher mean SWV observed in stiffer and more dense tissues [20]. The assessment of SWE as a marker of tissue fibrosis has been reported in various organs, including the placenta [15,19,21]. 

Although different forms of in vivo placental elastography techniques have been investigated [22,23,24,25,26], the evidence for their utility as a reliable tool for the assessment of placental function is limited and inconclusive [24,27,28,29,30,31,32,33,34,35,36]. The aim of this study was to prospectively investigate the relationship between placental SWV and fetal weight, and thus ascertain if it is a suitable proxy measure of placental function.

## 2. Materials and Methods

### 2.1. Design and Study Population

This was a prospective, observational, cohort study of women referred to the Mater Centre for Maternal and Fetal Medicine at the Mater Mother’s Hospital in Brisbane, Australia, between May 2022 and May 2023. All women provided informed consent for participation in the study. The study was conducted in accordance with the Declaration of Helsinki [37], with ethical and governance approvals provided by the Mater Misericordiae Limited Human Research Ethics Committee (HREC/MML/66263) and the Mater Governance Office, respectively. Administrative review approval was provided by the QUT Office of Research Ethics and Integrity (HE-AdRev 2024-8281-17583). 

Women with singleton SGA pregnancies [4] (EFW < 10th centile for gestation) ≥ 22 weeks’ gestation and without known aneuploidy, genetic syndromes, or major structural anomalies were invited to participate. Gestational age was calculated based on first trimester crown-rump length measurement. The referent population was women with pregnancies that were appropriate for gestational age (AGA) defined as EFW > 10th centile with normal fetoplacental Dopplers. All fetal growth and wellbeing ultrasound and SWE examinations were performed by a single sonographer (EC) in accordance with ISUOG practice guidelines [12,38] at 2–4-week intervals until birth, depending on advice from the treating Maternal Fetal Medicine specialist. EFW was calculated using Hadlock’s formula [39]. In addition to fetal biometry, the umbilical artery pulsatility index (UA PI), middle cerebral artery pulsatility index (MCA PI), cerebroplacental ratio (CPR), ductus venosus pulsatility index (DV PI), and uterine artery pulsatility index (UtA PI) were measured. Decisions regarding obstetric management and timing of the birth were independent of placental elastography findings.

### 2.2. Equipment and Methods

**Figure 1:**
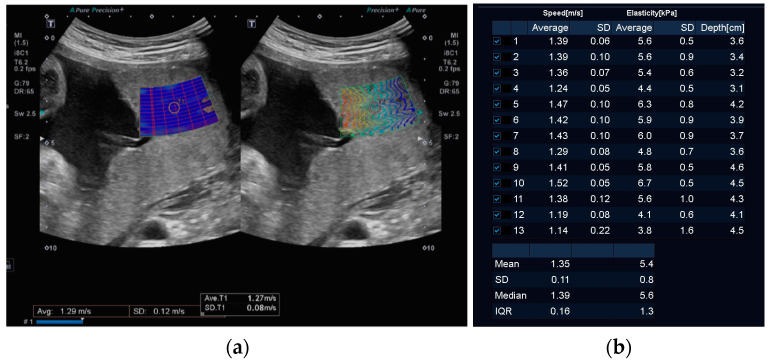
(**a**) The single 5 mm region of interest (ROI) placed in an area of good reliability as guided by the propagation map on the left; Homogeneous colour in the box on the left side of the image indicates uniform SWV, while on the right side, closely-spaced parallel lines indicate good reliability and low standard deviation. (**b**) Calculation of the mean, SD, median, and IQR from thirteen separate ROIs as recommended by the manufacturer.

SWE measurements were acquired using a Canon Aplio i-series 600 ultrasound system (Canon Medical, Otawara-shi, Japan) with a curved 8C1 transducer, as previously described [40]. Briefly, the transducer was positioned in a sagittal plane perpendicular to the placenta with the SWE acquisition field of view centrally within the placental parenchyma, as demonstrated in Figure 1. Measurements were obtained using the system’s single-shot method, which activates a 2.5 MHz acoustic radiation force pulse over a single frame. A 5 mm region of interest (ROI) was placed in an area of good reliability as guided by the propagation map. As recommended by the manufacturer, the mean SWV (m/s^2^) was calculated from thirteen separate ROIs [41]. Mean SWVs, where the standard deviation (SD) of the mean was higher than 10 percent, were discarded as this indicated poor reliability of SWV measurements [42,43]. If thirteen reliable or valid measurements could not be obtained due to impaired quality of SWV acquisition, the assessment was considered incomplete, and the participant was excluded from the study. As the depth of SWE samples is known to affect the reliability of the measurement, the average depth of the thirteen samples was also calculated and incorporated into the analysis [40]. No more than six single-shot SWE acquisitions were performed at any one examination, and the duration of the elastography examination did not exceed five minutes per examination. Care was taken to ensure no fetal parts were included in the SWE field. Thermal and mechanical indices were displayed on all acquisitions, and the thermal index did not exceed recommended limits at any time [44]. 

All data (maternal demographic, biomarker measurements, ultrasound measurements, and other relevant clinical details) were collected and managed using RADCap electronic data capture tools hosted at Mater Research Institute. REDCap (Research Electronic Data Capture) is a secure, web-based software platform designed to support data capture for research studies [45,46]. All healthcare providers were blinded to the SWV results, thus reducing potential bias, and any clinical decisions were made purely on the findings of the fetal growth and wellbeing assessment.

### 2.3. Statistical Analysis

The distribution of continuous variables was assessed using histograms. Maternal demographic characteristics were compared using Chi-square tests for categorical variables and either *t*-tests or Wilcoxon Rank Sum tests for continuous variables, according to distribution.

The unadjusted association between mean SWV and EFW was initially assessed with locally estimated scatterplot smoothing (Lowess) to fit a smooth curve through the scatterplot of data. The correlation between SWV and EFW was assessed using Spearman’s rank correlation coefficients, with correlations considered significant at *p* < 0.05. Multiples of the median (MoMs) were calculated for SWV, adjusting for gestational age and average tissue depth at examination. Multiples of median were used rather than z-scores because SWV was not normally distributed.

Multivariable logistic regression was used to investigate the relationship between MoMs for SWV and SGA, adjusting for confounders based on clinical plausibility. There were no clinically relevant effect measure modifiers to consider. Variables were categorized if the assumption of linearity between continuous variables and the log odds of SGA was violated. MoMs for SWV were categorized into (1) quartiles, (2) deciles, (3) a binary variable of the highest quartile, and (4) a binary variable of the highest decile. Models accounted for clustering at the patient level due to repeated observations. Goodness of fit was confirmed by the Hosmer–Lemeshow goodness-of-fit test. A multiple-record format was used to enable SWE to be analyzed at multiple time points throughout pregnancies. Risk estimates were presented as odds ratios (ORs) and 95% confidence intervals (95% CIs).

As there are no previous studies investigating the association between SWV and EFW, sample size calculations were not performed. Significance was set at α = 0.05 for all statistical tests. The reporting of this study conforms to the STROBE (Strengthening the Reporting of Observational Studies in Epidemiology) statement [47]. Statistical analyses were performed using Stata 18^®^ (Statacorp LLC, College Station, TX, USA).

## 3. Results

### Patient Demographics and Inclusion Criteria

**Figure 2:**
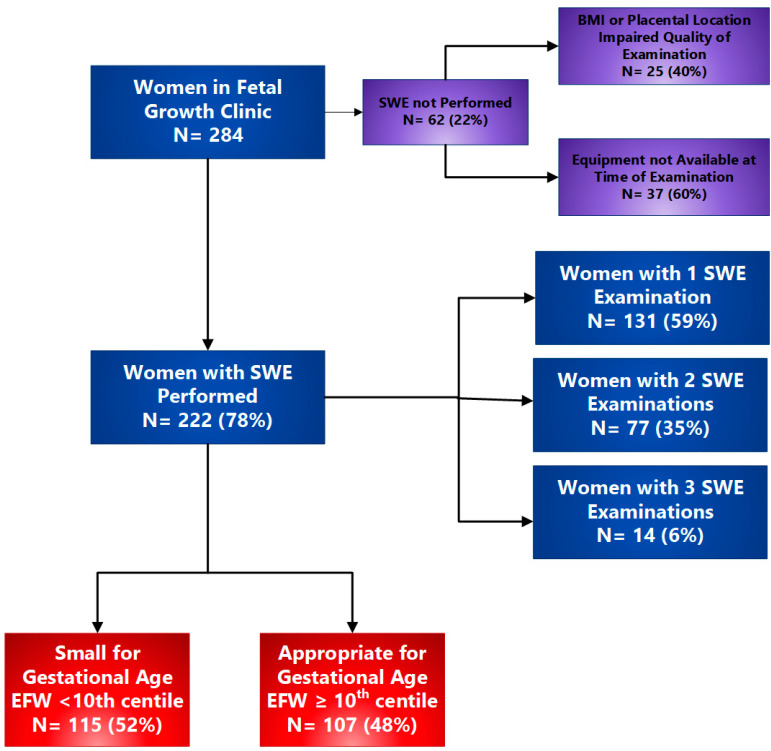
Study flow chart. SWE: shear wave elastography; BMI: body mass index; EFW: estimated fetal weight.

Over the study period, 284 women met the inclusion criteria and were recruited into the study. SWE was unable to be performed on 62 women because either maternal BMI or placental location and depth precluded adequate assessment of the placenta, leaving 222 women on whom SWE was successfully performed, thus constituting the final study cohort (Figure 2). 

Women with SGA pregnancies comprised 52% (115/222) of the study cohort. Of these, 131 (59%) had one, 77 (35%) had two, and 14 (6%) had three SWE assessments. Table 1 lists the demographic and health characteristics of the study population. Women in the SGA cohort were more likely to be nulliparous, have a lower BMI, be of South Asian ethnicity, and have pre-eclampsia.

**Figure 3:**
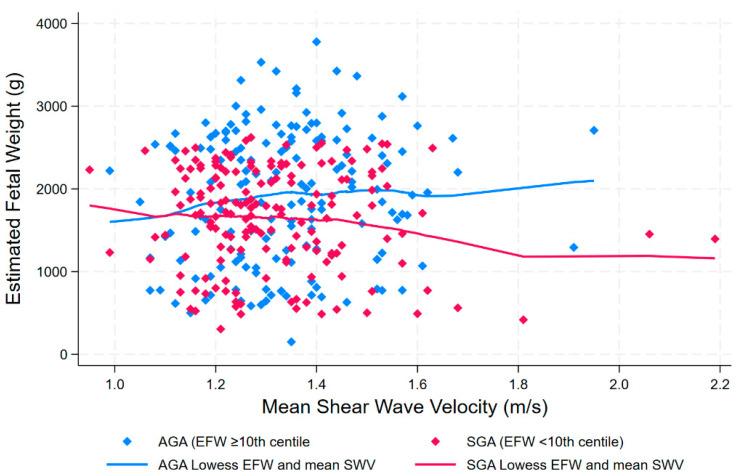
Scatterplot and locally estimated scatterplot smoothing (Lowess) curve investigating unadjusted relationship between estimated fetal weight and shear wave velocity in appropriate-for-gestational-age (AGA) and small-for-gestational-age (SGA) pregnancies.

Scatterplots and Lowess curves confirmed that there was no apparent relationship between mean SWV and EFW in either SGA or AGA pregnancies (Spearman’s rho 0.04, *p* = 0.51) (Figure 3). 

**Figure 4:**
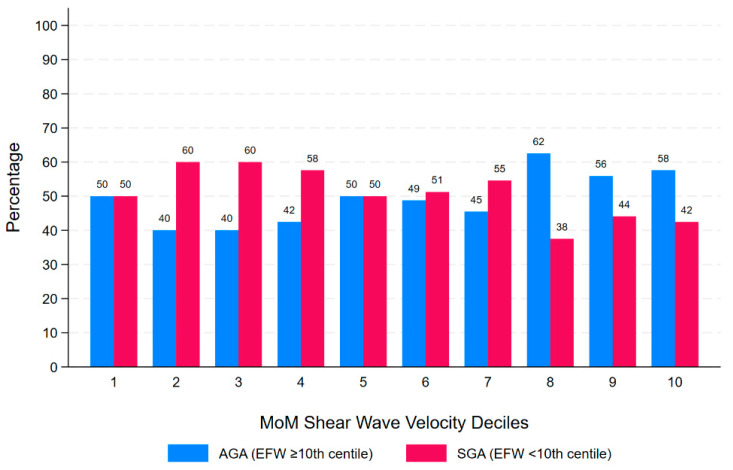
MoM SWV deciles, adjusted for gestational age at examination and average depth at examination, and percentage of pregnancies within each decile that are AGA (EFW ≥ 10th centile) and SGA (EFW < 10th centile). AGA: appropriate for gestational age; SGA: small for gestational age; EFW: estimated fetal weight.

Multivariable logistic regression models developed to investigate the association between SWV and SGA, adjusting for BMI, showed that SWV MoM was not associated with EFW < 10th centile in any model, regardless of whether SWV MoM was examined as quartiles, deciles, or binary variables of the highest quartile and deciles (Table 2). Figure 4 presents the deciles of SWV MoM and the percentage of each decile for EFW < 10th centile and controls.

## 4. Discussion

The results of this large prospective study show that there is no difference between placental SWV in SGA compared to AGA pregnancies. In addition, we did not find any correlation between placental SWV and EFW. Our results, therefore, suggest that the assessment of placental SWV may have limited clinical value in pregnancies complicated by placental dysfunction.

Although recent systematic reviews [21,48,49] evaluating the utility of SWE for the assessment of placental function concluded that SWE is an objective and repeatable method of quantitatively assessing placental tissue, its clinical role is still unclear. Indeed, our findings contrast with some other studies showing a higher placental SWV in SGA pregnancies [28,29,50]. This may in part be explained by limitations in measurement accuracy and precision in the performance of SWE, as outlined in the review by Saw et al. [34]. The authors of that review highlighted the need for large properly validated elastography datasets that utilize appropriate methodology to obtain SWV. Compared to other similar studies, our study cohort of SGA pregnancies is one of the largest of its kind, underlining the robustness of our findings [28,29,50].

Another strength of our study is the reliability of the SWV measurements we obtained. Like any ultrasound-derived quantitative measurement, such as Doppler velocimetry, SWE is susceptible to measurement variability. Inconsistent shear wave measurements may be caused by an increased depth of the sample, attenuation of the overlying tissues, or operator-related variations [43]. The review of umbilical–placental biomechanics performed by Saw et al. [34] found that although non-invasive placental elastography could be useful in defining placental disease, the measurement accuracy was highly dependent on a controlled setting. Our SWE measurements were performed by a single experienced sonographer adhering to a strict protocol. An earlier paper from our group [43] showed that for a large inhomogeneous organ such as the placenta, accurate assessment of tissue elasticity requires a larger number of small ROIs. Our study utilized the mean SWV of thirteen separate 5 mm ROIs. Additionally, SWV ROI measurements were rejected or remeasured from the propagation map if the SD of the mean was found to be greater than 10 percent. Our study’s rigor ensured a high standard of reliability and reproducibility. This is an important factor in elastographic evaluation of the placenta and other organs as previous studies have shown wide variation in their results and high user dependency [18,34,42,51]. These limitations probably account for the wide variation in SWV measurements (up to 40%) in comparable studies [27,28,33,50,52].

Unlike previous similar studies which only examined placentae that were located on the anterior uterine wall [28,33] (presumably for ease of access), our study found that reliable measurements could be achieved from lateral, fundal, and sometimes posteriorly sited placentae. Some placental SWE studies reported high rates (~40%) of unfavorable placental location of the study population, and thus, exclusion of these participants significantly reduced the size of the study cohort [53]. We found that although the quality of the SWE sample (as judged by the propagation map and SD) was influenced by the depth of acquisition, many of these non-anterior placentae were often ≤8 cm in depth away from the transducer and therefore technically accessible [19,43,54]. As SWE is sensitive to sampling depth [43,55,56], we adjusted for average tissue depth. Other publications do not appear to have made similar adjustments, which may be another reason for the discrepancy in study findings [28,50,57,58].

We recognize that there are limitations of this study, the first being that it was conducted in a single center. Secondly, we only focused on pregnancies complicated by SGA growth and not necessarily those with overt FGR [59]. Indeed, there was no difference in the median umbilical artery pulsatility index between the cohorts, suggesting that the severity of placental dysfunction in the SGA cohort may not have been substantial. Future work should perhaps focus on pregnancies affected by overt fetal growth restriction and incorporate other measures of placental function such as placental biomarkers (e.g., placental growth factor). Additional placental assessment, such as histopathology and volumetric measurements, may also complement the measurement of placental mechanical properties with SWE and assist us in better understanding placenta-mediated fetal growth abnormalities.

## 5. Conclusions

The detection and management of suboptimal fetal growth resulting from placental underperfusion is a major challenge for clinicians. Current practice relies on ultrasound biometry and Doppler velocimetry as indirect assessments of placental function. Although SWE is a novel, ultrasound-derived method of estimating tissue stiffness, its utility for placental assessment remains to be proven, unlike its use in assessing liver fibrosis and characterizing breast and thyroid lesions [17,20]. Although some previous studies have shown modest potential for SWE in pregnancies complicated by putative placental dysfunction causing perturbation in fetal growth [24,27,28,29,33,50], the results from our study show potentially limited utility for the assessment of SGA placentae. 

## Figures and Tables

**Table 1:** Maternal demographic, health, and ultrasound-derived parameters stratified for small-for-gestational-age pregnancy.

	Total	EFW ≥ 10th Centile	EFW < 10th Centile	*p*-Value
	*n* = 222	*n* = 107	*n* = 115	
Gestational age	35 (32, 36)	35 (31, 36)	35 (33, 36)	0.66
Age	31 ± 6	31 ± 6	32 ± 6	0.85
Nulliparous	86 (38.7%)	32 (29.9%)	54 (47.0%)	0.009
Body mass index at recruitment (kg/m^2^)	25 (21, 31)	25 (22, 31)	25 (21, 30)	0.10
Mean arterial pressure at recruitment (mmHg)	82 (77, 88)	82 (77, 87)	82 (77, 89)	0.22
Ethnicity				0.008
Nth and Sth American, Australia, NZ, and Europe	105 (47.3%)	52 (48.6%)	53 (46.1%)	
NE, SE Asian, and Oceana ^1^	43 (19.4%)	14 (13.1%)	29 (25.2%)	
South Asian	40 (18.0%)	16 (15.0%)	24 (20.9%)	
African	29 (13.1%)	20 (18.7%)	9 (7.8%)	
Middle Eastern	2 (0.9%)	2 (1.9%)	0 (0.0%)	
Other or mixed ethnicity	3 (1.4%)	3 (2.8%)	0 (0.0%)	
Indigenous	15 (6.8%)	8 (7.5%)	7 (6.1%)	0.43
Spontaneous conception	205 (92.3%)	101 (94.4%)	104 (90.4%)	0.16
Smoking during pregnancy	21 (9.5%)	7 (6.5%)	14 (12.2%)	0.15
Alcohol during pregnancy	3 (1.4%)	1 (0.9%)	2 (1.7%)	0.60
History of SGA baby	73 (32.9%)	36 (33.6%)	37 (32.2%)	0.82
Connective tissue disease in pregnancy	4 (1.8%)	2 (1.9%)	2 (1.7%)	0.94
Renal disease in pregnancy	3 (1.4%)	2 (1.9%)	1 (0.9%)	0.52
Diabetes in pregnancy	65 (29.3%)	30 (28.0%)	35 (30.4%)	0.69
Hypertension in current pregnancy	43 (19.4%)	18 (16.8%)	25 (21.7%)	0.35
Pre-eclampsia in current pregnancy	36 (16.2%)	9 (8.4%)	27 (23.5%)	0.002
Low-dose aspirin use	62 (27.9%)	38 (35.5%)	24 (20.9%)	0.015
Ultrasonographic Parameters				
Mean shear wave velocity—SWV (m/s) ^2^	1.30 (1.21, 1.41)	1.33 (1.24, 1.41)	1.27 (1.20, 1.40)	0.05
Standard deviation of SWV (m/s) ^2^	0.12 (0.11, 0.15)	0.12 (0.11, 0.14)	0.12 (0.10, 0.15)	0.70
Average depth (cm) ^2^	4.49 (3.75, 5.30)	4.53 (3.73, 5.42)	4.46 (3.90, 5.18)	0.59
Umbilical artery EDF ^2^				0.008
Positive EDF	300 (97.1%)	158 (100.0%)	142 (94.0%)	
Absent EDF	6 (1.9%)	0 (0.0%)	6 (4.0%)	
Reversed EDF	3 (1.0%)	0 (0.0%)	3 (2.0%)	
Umbilical artery pulsatility index ^2^	0.98 (0.85, 1.11)	0.97 (0.86, 1.10)	1.00 (0.85, 1.14)	0.15
Estimated fetal weight ^2^	1808 (1223, 2347)	2003 (1212, 2582)	1707.50 (1231, 2206)	<0.001

Data are presented as mean ± SD or median (IQR) for continuous measures, and *n* (%) for categorical measures. ^1^ Excluding Australian and New Zealand; ^2^ *n* = number of observations (examinations) rather than number of patients. EDF: End Diastolic Flow.

**Table 2:** Multivariable logistic regression models investigating the association between multiples of the median for shear wave velocity (MoM SWV) and small for gestational age (SGA), defined as estimated fetal weight < 10th centile. ^a^ Multivariable regression models adjusted for body mass index. ^b^ MoM adjusted for gestational age at examination and average tissue depth at examination.

Model		Univariable OR (95% CI)	*p*-Value	Multivariable ^a^ OR (95% CI)	*p*-Value
1	MoM SWV ^b^ Quartiles				
	1 (Lowest)	referent		referent	
	2	1.07 (0.59, 1.96)	0.82	1.06 (0.58, 1.94)	0.85
	3	0.77 (0.42, 1.39)	0.38	0.78 (0.43, 1.43)	0.43
	4	0.64 (0.34, 1.22)	0.18	0.67 (0.35, 1.29)	0.23
2	MoM SWV ^b^ Deciles				
	1 (Lowest)	referent		referent	
	2	1.50 (0.52, 4.31)	0.45	1.50 (0.53, 4.27)	0.44
	3	1.50 (0.55, 4.08)	0.43	1.45 (0.53, 3.94)	0.47
	4	1.36 (0.51, 3.62)	0.54	1.41 (0.52, 3.79)	0.50
	5	1.00 (0.35, 2.82)	1.00	1.00 (0.36, 2.77)	0.99
	6	1.05 (0.41, 2.68)	0.92	1.06 (0.42, 2.71)	0.90
	7	1.20 (0.45, 3.23)	0.72	1.25 (0.46, 3.42)	0.66
	8	0.60 (0.22, 1.61)	0.31	0.62 (0.23, 1.66)	0.35
	9	0.79 (0.3, 2.08)	0.63	0.84 (0.32, 2.24)	0.73
	10	0.74 (0.27, 2.03)	0.56	0.77 (0.28, 2.11)	0.61
3	MoM SWV ^b^ Highest Quartile				
	Quartiles 1–3	referent		referent	
	Highest Quartile	0.69 (0.41, 1.17)	0.17	0.73 (0.43, 1.23)	0.24
4	MoM SWV ^b^ Highest Decile				
	Deciles 1–9	referent		referent	
	Highest Decile	0.69 (0.32, 1.48)	0.34	0.71 (0.33, 1.52)	0.38

## Data Availability

The data presented in this study are available on request from the corresponding author provided that appropriate ethics approval, inter-institutional data sharing agreements, and other regulatory requirements are in place.
